# Prevalence of knee injuries among male college students in Riyadh, Kingdom of Saudi Arabia

**DOI:** 10.1186/s13018-020-01638-1

**Published:** 2020-03-31

**Authors:** Abdulaziz Almaawi, Waleed Awwad, Azzam Bamugaddam, Muath Alasheikh, Mohammed Muaddi, Omar Almutair, Abdulaziz Z. Alomar

**Affiliations:** 1 Orthopedic Surgery Department, College of Medicine, King Saud University, Riyadh, Saudi Arabia; 2 Dr. Sulaiman Al Habib Medical Group, Riyadh, Saudi Arabia; 3 Orthopedic Surgery Department, King Abdulaziz University, Jeddah, Saudi Arabia

**Keywords:** Knee injuries, Prevalence, Saudi Arabia

## Abstract

**Background:**

The knee is considered the most common injured joint between young sport participants. However, there is lack of proper prevalence estimation in our population.

The purpose of this study was to identify the prevalence of knee injuries among male college students and to observe the demographic data associated with it. Our secondary objective was to evaluate the awareness and knowledge about these injuries.

**Methods:**

This is a cross-sectional study. A survey was distributed to collect the data among male college students, King Saud University, Riyadh, Saudi Arabia. Out of 688 students who participated and completed the questionnaire, a total of 482 were considered valid and met the inclusion criteria. Data were analyzed using Statistical Package for Social Sciences (SPSS).

**Results:**

The overall prevalence of knee injury was 23.2% (*n* = 112). Most of them injured during sport activities especially soccer and 68.7% involved in a non-contact mechanism of injury. Among those who went to a hospital mostly were diagnosed as contusion (31.4%) then as meniscus tear, ACL, and collateral ligament injury, respectively. Majority was treated conservatively and only 10.7% needed surgery surprisingly. There was no statistically significant difference between those who are injured and whether they were warmed up and stretched or not (*P* = 0.619). Low level of knowledge about knee injuries was noticed among the participants 57.7%.

**Conclusion:**

Our study has highlighted the high prevalence of knee injuries and the need to raise the level of awareness and knowledge about these injuries in our population. Soccer was the most common sport associated with knee injuries; most of these injuries were treated conservatively.

## Introduction

The knee is the largest joint in the body and a very complex structure. And the most commonly injured joint by young sports participants [1, 2]. The vast majority of knee injuries can be treated conservatively with rest, ice, immobilization, and physiotherapy. However, others might require surgical intervention.

Knee injuries could be either acute or chronic. Acute knee injuries by most definitions are defined as “being diagnosed within the first 30 to 42 days of the injury or onset of symptoms.”

Knee injuries range from ligamentous to cartilaginous, tendinous, and bony injuries. A detailed history with physical examination and proper investigations is crucial to make the right diagnosis. Most of the knee injuries occur in a non-contact manner involving mainly the anterior cruciate ligament (ACL) which is the major stabilizer of the joint and mostly accompanied by another structural injury [3–8]. Other ligaments can be involved, including the posterior cruciate ligament (PCL), lateral collateral ligament (LCL), and medial collateral ligament (MCL). Moreover, cartilaginous structure where injury to the medial and lateral menisci—that act as shock absorbers with a secondary role in stabilizing the joint—can be torn or partially involved [9].

A major complication following most of the knee injuries is the early development of osteoarthritis where long term management is expected [10, 11].

At King Fahd University Hospital, Al Khobar, Eastern Province, Saudi Arabia, a prospective study of sports-related injuries was conducted in a period of 12 months and knee injuries represented the majority of injuries encountered with a 27% [12]. Soccer is the leading sport activity associated with knee injuries [12, 13]. In the USA, most of the collegiate studies were concerned with knee injuries among athletes; such an epidemiological study was conducted in the National Collegiate Athletic Association (NCAA) among men’s and women’s volleyball athletes where the knee joint was the most commonly injured body part (25.5% in males; 16.3% in females), with different patterns of injuries between male and female [14].

Strengthening and stretching exercises have been described to have a role in preventing anterior knee pain, where increasing the flexibility of a muscle-tendon unit can promote a better performance and might have a role in decreasing the occurrence of injuries [15–18]. However, a systematic review showed no benefit of stretching exercises, where strengthening exercises had marked reduction by 50% in overuse injury. Therefore, conducting well-controlled randomized trials is needed to figure out the exact role of stretching in sports and a better understanding of the contradictory findings that have been reported in the literature [19–22].

The prevalence of knee injuries in our population lacks proper estimation, especially, nowadays with wide variety of sporting facilities available for the youth to practice all different types of sports which will increase the likelihood of sport-related injuries. The aim of this study is to estimate the prevalence of knee injuries among Saudi males and their level of awareness of such injuries and initial management. In our population, females are less involved in sporting events and access to female college students is not feasible as compared to males.

## Methodology

### Study design and subjects

A cross-sectional study was conducted to estimate the prevalence, risk factors, knowledge, and practice toward knee injuries among male college students in Riyadh, Saudi Arabia, in 2018. A hard copy questionnaire was distributed among 706 students, 688 of whom completed the questionnaire, giving a response rate of 97.5%.

### Inclusion and exclusion criteria

The following inclusion criteria were then used: (1) minimum age of 18 years, (2) a Saudi male, (3) a college student, (4) and a written informed consent. Any subjects with a congenital deformity or a degenerative disease were excluded from the study. The data were collected using a structured, self-administered questionnaire meeting the objectives of the study.

All participants signed a written informed consent explaining the purpose of the study, reassuring them regarding the confidentiality and privacy of their data.

### Questionnaire and variables

The questionnaire was designed by the researchers after reviewing the literature and similar questionnaires. Some linguistic and technical modifications were made. The questionnaire was then reviewed, and the study was approved by the Institutional review board (IRB) in the College of Medicine, King Saud University, Riyadh, Saudi Arabia.

The questionnaire consisted of five parts: (1) sociodemographic data: age, weight, height, health status, activity level, hours of training weekly, and history of knee injury; (2) knee injuries-related questions including mechanism of injury, prior warm up and stretching, weather sport-related or not, time elapsed since last injury, applied type of treatment, and if any knee structures damaged during the injury; (3) pain assessment and associated symptoms: severity on a scale from 1–10, associated with activity or rest, affected range of motion (ROM), and ambulatory aids; (4) quality of life: knee injury and osteoarthritis outcome score (KOOS) for quality of life was calculated from 0 (worst) to 100 (best), and (5) awareness, knowledge, and practice.

**Fig. 1:**
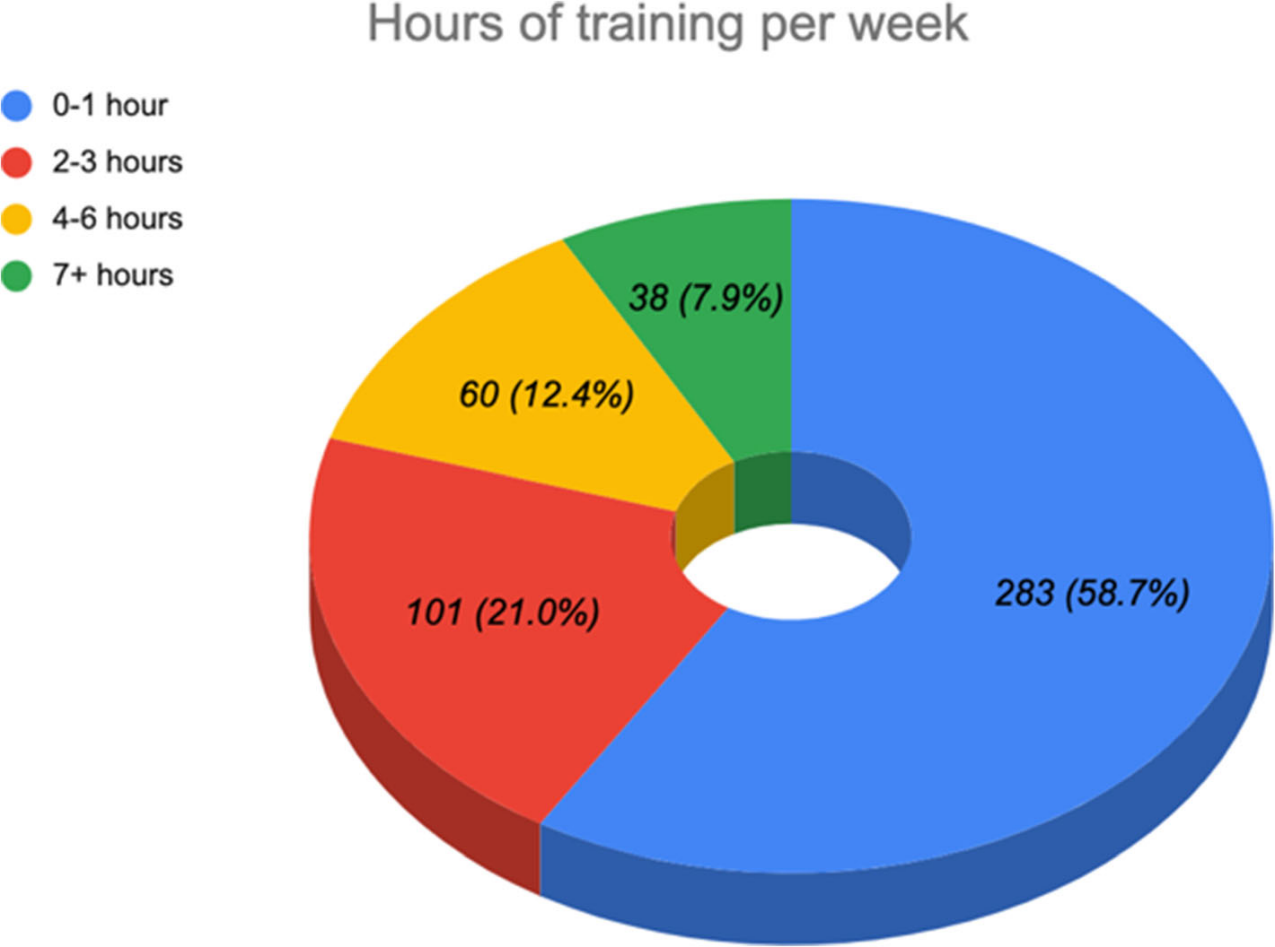
Hours of training per week

Body mass index (BMI) was classified into six categories according to the World Health Organization classification: < 18.5 as underweight, 18.5–24.9 as normal weight, 25–29.9 as overweight, 30–34.9 as obesity class I, 35–39.9 as obesity class II, and ≥ 40 as obesity class III. Age was classified into two groups, 18–21 and 21–25. Health status was classified into five categories: poor, fair, good, very good, and excellent. Activity level was subdivided into four categories: non-sporting, sporting sometimes, well-trained and frequently sporting, and highly competitive sports person. Hours of training per week were distributed into four intervals 0–1, 2–3, 4–6, and + 7 (Fig. 1). A score to assess the knowledge level was adapted based on the participant’s answers to a list of nine questions about basic anatomy and its function in addition to the investigations used to diagnose a knee injury: high ≥ 5 and low < 5. Correlation in sport participants between warm up/stretching and the incidence of knee injury was tested also.

### Data analysis

Completion and return of the questionnaire were considered as an absolute to participation in the study. Returned questionnaires that did not meet the inclusion criteria were excluded, giving a total of 482 valid questionnaires. The data were coded, entered, and analyzed using Statistical Package for Social Sciences (SPSS)

Data were presented using descriptive statistics in the form of frequencies, percentages for categorical variables, mean, and standard deviation for quantitative variables. Chi-square test was used to test any correlation.

## Result

A total of 688 students responded to the survey (97.5% response rate). Four hundred eighty-two respondents met the inclusion criteria and were included in the analyses.

The overall prevalence of knee injury was 23.2% (*n* = 112). One hundred one of them were due to sport activities. Two hundred fifty-nine of the participants (53.73%) were in the age group of (18–21), with the prevalence of knee injury highest at 25 years of age 39.4% (*n* = 26) (*P* = 0.030).

Among 482 students exhibited 21.54 ± 2.09 years (*P* = 0.005), 173.79 ± 7.32 cm of height, and 80.05 ± 21.96 kg of body mass. Majority of the students had a normal BMI *n* = 210 (43.6%) with the highest prevalence of knee injury in class II obese individuals *n* = 9 (29%) (*P* = 0.025). Most of the students described their health status as “Very Good” (36.72%) with regular period of training in the range of 0–1 h per week (58.71%).(46.89%) of the subjects described their activity level as “Sporting sometimes”, with the highest prevalence of knee injury in “well-trained and frequently sporting” individuals (35.6%) (*P* = 0.004). The majority exhibited a “Low” knowledge level—which was explained in the methodology section—*n* = 277 (57.5) (Table 1).

**Table 1:** Distribution of study participants according to demographic profile, body mass index, activity level, and knowledge

Variable	Frequency (%) ***n*** = 482 (100%)	Have you ever had a knee injury?		***P*** value
		Yes ***n*** = 112 (23.2%)	No ***n*** = 370 (76.8%)	
**Age**				
18	36 (7.5)	5 (13.9)	31 (86.1)	
19	48 (10)	9 (18.8)	39 (81.3)	
20	79 (16.4)	17 (21.5)	62 (78.5)	
21	96 (19.90)	22 (22.9)	74 (77.1)	0.030
22	65 (13.50)	15 (23.1)	50 (76.9)	
23	62 (12.90)	9 (14.5)	53 (85.5)	
24	30 (6.22)	9 (30.0)	21 (70.0)	
25	66 (13.70)	26 (39.4)	40 (60.6)	
Mean ± SD	21.54 ± 2.09	22.02 ± 2.19	21.4 ± 2.04	0.005
**Age groups**				
18–21 years	259 (53.73)	53 (20.5)	206 (79.5)	0.120
22–25years	223 (46.27)	59 (26.5)	164 (73.5)	
**Weight** (mean ± SD)	80.05 ± 21.96	81.97 ± 22.07	79.47 ± 21.92	0.293
**Hight** (mean ± SD)	173.79 ± 7.32	174.98 ± 6.27	173.44 ± 7.58	0.23
**BMI classifications**				
Underweight	35 (7.3)	3 (8.6)	32 (91)	
Normal	210 (43.6)	55 (26.2)	155 (73.8)	0.025
Overweight	125 (25.9)	35 (28)	90 (72)	
Obese I	59 (12.2)	7 (11.9)	52 (88.1)	
Obese II	31 (6.4)	9 (29)	22 (71)	
Obese III	22 (4.6)	3 (13.6)	19 (86.4)	
Mean ± SD	26.37 ± 6.8	26.31 ± 5.96	26.39 ± 7.05	0.511
**Obesity**				
Yes	112 (23.2)	19 (17)	93 (83)	.075
No	370 (76.8)	93 (25.1)	277 (74.9)	
**Health status**				
Poor	11 (2.28)	2 (18.2)	9 (81.8)	0.584
Fair	26 (5.39)	8 (30.8)	18 (69.2)	
Good	102 (21.16)	28 (27.5)	74 (72.5)	
Very good	177 (36.72)	36 (20.3)	141 (79.7)	
Excellent	166 (34.44)	38 (22.9)	128 (77.1)	
**Activity level**				
Non-sporting	138 (28.63)	25 (18.1)	113 (81.9)	0.004
Sporting sometimes	226 (46.89)	45 (19.9)	181 (80.1)	
Well-trained and frequently sporting A high competitive sports person	101 (20.95) 17 (3.53 )	36 (35.6) 6 (35.3)	65 (64.4) 11 (64.7)	
**How many hours per week, do you train**				
0–1	283 (58.71)	59 (20.8)	224 (79.2)	0.118
2–3	101 (20.95)	22 (21.8)	79 (78.2)	
4–6	60 (12.45)	21 (35.0)	39 (65.0)	
7 +	38 (7.88)	10 (26.3)	28 (73.7)	
**Knowledge level**				
High	205 (42.5)	41 (20)	164 (80)	0.148
Low	277 (57.5)	71 (25.6)	206 (74.4)	

In the past 12 months, 48 injuries out of 112 occurred (42.9%). When asked about the mechanism of injury, 68.7% (*n* = 77) of the participants were involved in a “non-contact injury.” Fifty-one percent (*n* = 57) of the injured went to the hospital and the diagnosis was reported as 31.4% (*n* = 18) contusion, 17.5% (*n* = 10) meniscus tear, 14.3% (*n* = 10) ACL, 3.5% (*n* = 2) PCL, 12.5% (*n* = 10) collateral, 3.5% (*n* = 2) fractures, 1.8% (*n* = 1) patellar dislocation, and 14% (*n* = 8) do not know the diagnosis. Sixty-one injuries (55.5%) needed medical attention, 20 (17.9%) demanded physiotherapy, and 12 (10.7%) were subject to surgery. Only 17.9% (*n* = 20) reported the need for using a knee protection during activities and 9.28% (*n* = 9) required ambulatory aids for the pain. In terms of time needed for recovery, 17.9% (*n* = 20) needed 1–3 days, 19.6% (*n* = 22) needed 4–7 days, 17% (*n* = 19) needed 1–4 weeks, and 45.5% (*n* = 51) needed more than 4 weeks. Regarding the pain, 62.5% (*n* = 70) described their pain as intermittent, 24.1% (*n* = 27) as constant, and 13.4% (*n* = 15) reported no pain at all, and the mean of pain severity was 6.05 ± 2.41, 84.54% (*n* = 82) said their pain was associated with activity, 61.86% (*n* = 60) were able to straighten their knee without any pain, and 49.48% (*n* = 48) were able to bend their knee without pain. Only 7.22% (*n* = 7) received injection to relieve their pain. Among the injured, the overall prevalence of knee pain was 86.8%. The second most common symptom in relation to the knee joint was hearing “popping sound” 75.9%, followed by “hanging up when moving” 50%, and “swelling” 46.4%.

Among the injured, soccer was contributing to most injuries (*n* = 85), followed by strength training (*n* = 7), volleyball (*n* = 3), running (*n* = 3), biking (*n* = 1), skydiving (*n* = 1), and karate (*n* = 1). The majority of the participants chose “warm up and stretching” as a preventive measure *n* = 354 (73.4%). On the other hand, most of them believed that “proper equipment,” “body awareness,” and “training and conditioning” have no role in preventing sports-related injuries. A total of 35.6 % of the injured were warming up and stretching before exercising (*n* = 36). When this preventive measure warming up and stretching prior to any sporting activity were tested, there was no statistically significant difference between those who are injured and whether they were warming up and stretching or not (Table 2).

**Table 2:** Frequency of injures per type of sport and either warming up and stretching was done or not

Variable	Frequency (%) (***n*** = 101) (100%)	Warming up and stretching before sport		***P*** value
		Yes ***n*** (%) 36 (35.6)	No (***n*** = 65) (64.4)	
Soccer	85 (84.2)	29 (34.1)	56 (65.9)	0.619
Volleyball	3 (3)	1 (33.3)	2 (66.7)	
Strength exercise	7 (6.9)	4 (57.1)	3 (42.9)	
Biking	1 (1)	0 (0)	1 (1)	
Skydiving	1 (1)	0 (0)	1 (1)	
Running	3 (3)	1 (33.3)	2 (66.7)	
Karate	1 (1)	1 (1)	0 (0)	

When encountering a knee injury in the future, *n* = 271 (56.2%) chose not to put “ice,” *n* = 437 (90.7%) said “no” to raising the injured limb, *n* = 393 (81.5%) would not take pain killers, and *n* = 404 (83.8%) said “no” to applying bandage to the injured knee.

Our population knowledge was assessed by adapting a score based on a list of simple nine questions concerned with the basic anatomy, muscles and movements, and types of investigations used to diagnose a knee injury, *n* = 277 (57.5%) displayed a “low” score (Table 1).

## Discussion

In this study, we explored different aspects of knee injuries including its prevalence, associated factors, and awareness on male collegiate students. The prevalence of knee injuries was 23% among participants. This is higher than what was reported 13.5% on a comparable study done on college students in Delhi, India [23]. However, the prevalence we found still falls within the global prevalence range among adolescent between 10 to 25% based on a systematic review done by Louw et al. that included 19 studies, with more recent studies reporting higher rates [24]. At the University of Central Lancashire, Preston, UK, The prevalence of knee injuries was estimated 31.8% among their students with knee pain as the predominant symptom [25].

Although our sample age ranges from 18 to 25 years old, it was found that more than half of them practicing sport for 1 h or less per week. This reflects a sedentary life which is prevalent in this part of the world. In comparison with the Physical Activity Guidelines for Americans from the US Department of Health and Human Services, this age group should be more active. They stated, “Adults should do at least 150 to 300 min a week of moderate-intensity, or 75 to 150 min a week of vigorous-intensity aerobic physical activity.” Therefore, for someone to be considered physically active and obtain significant health benefits should do at least 2 h and a half of moderate-intensity level or 1 h and 15 min of vigorous-intensity level of aerobic exercises each week [26].

Participants who are involved more in sports, especially competitive ones were associated with a higher incidence of knee injuries. Our result is similar to the result of previous studies from the USA and the UK, which demonstrated an increased incidence of these injuries with increased level of competition [27, 28]. The most common mechanism led to these injuries was due to a non-contact mechanism (68.7%); this is consistent with what was observed by John et al. (64.4%) of their sample [13]. Furthermore, The American Journal of Sports Medicine reported by Arendt and Dick that non-contact mechanism was the reason for most ACL injuries among college basketball and soccer players in the USA [29].

We found association between increased body mass index (BMI) and prevalence of knee injuries, this result is in agreement with previous studies demonstrated increased risk of sport injuries in general and specifically knee injuries with increased BMI, and this can be attributed to the increased mechanical load on knee joints while doing sports or other physical activity [30–32]. Knee pain was the most frequent complaint followed by hearing a popping sound, knee catching, and swelling. Only half of the students went to the hospital at the time of their injury and most of them were diagnosed as contusion, followed by meniscus, and ACL injuries respectively. In comparison with prior studies, sprain was the most common diagnosis of knee injuries among collegiate footballers in the USA [33–35], but their classification was different, all types of ligamentous injuries were included under knee sprain. For example, ACL and MCL both classified under sprain, so the comparison here is inaccurate.

Among those who sought medical attention, most were treated conservatively by analgesia, physical therapy, and knee braces. Only 10% of participants underwent surgery. Similarly, studies by Nielsen et al. and Swenson et al. conservative treatment were the cornerstone of the management, but the percentage of surgical intervention was higher (20–21%) [36, 37].

Regarding knee braces and their effect, in a review paper conducted by Chew KT and colleagues [38], they described the different types and aspects of these braces. Several studies investigated the effect of patellofemoral knee braces, which are made to maintain a normal alignment of patella and alleviate anterior knee pain. But the results of these studies are inconsistent, some reported significant improvement on knee pain and function [39, 40], while others reported as insignificant [41, 42]. In Chew et al.’s review, they mentioned as there are different causes of knee pain some patients may have benefited, while others may not; they stated the need for further studies to investigate the effect of these braces on specific knee problems.

For the functional brace which is designed to support the knee after a ligamentous injury, Swirtun et al. published a prospective randomized study to evaluate the effect of functional bracing on patients with ACL tear who were treated non-operatively, patients reported significant improvement on rehabilitation and stability of their knees. However, these benefits were not evident on the objective measures including Knee Osteoarthritis Outcome Score (KOOS) and Cincinnati knee score, and only considered as subjective outcomes [43]. Wojtys et al. did a biomechanical study on using functional brace on ACL deficient knee and it showed significant decrease in anterior tibia translation [44]. In addition, the effectiveness of knee braces as a prophylactic measure, Hewson et al. has showed in a college football team at the university of Arizona in a prospective case control study and they found that the number of knee injuries was similar on both braced and non-braced groups [45]. On the other hand, other studies reported a higher incidence rate of knee injuries after using prophylactic knee braces [46].

Different types of activities were associated with knee injuries including strengthening exercises, volleyball, running, and different other sports but the majority of knee injuries were during soccer, which is consistent with a previous study done by Kujala et al. [47], but we cannot decide if it carries the highest risk for knee injuries without comparing the exposure hours for each sport, then we could know the injury rate and risk for each. So, this could be attributed to either the popularity of this sport in our culture and hence the percentage will be obviously higher or to the functional demand on the knee structures and movements that is required in this sport.

The prevailing idea in our population about how to prevent related sport injuries is by warming up and stretching exercises. About one-third of the participants with knee injuries were warming up and stretching before they start their sporting activities and there was no statistically significant difference between those who are doing this as a preventive measure and those who are not. A systematic review done by Lauersen et al. [19] supports our result of no benefit from stretching. Also, another systematic review is consistent with this result [21].

We found a lack of basic information about knee structures and how participants should deal with knee injuries if experienced in the future. Hence, indicating the necessity of health education about these common injuries and how to deal with them [48].

In the present study, 45% of participants needed more than 4 weeks to recover after their knee injuries and they were unable to return to their level of activity prior to the injury, others needed less time to recover. In comparison with other studies that reported decrease in the ability of some patients to carry out their previous level of physical activity even after they were cleared by their healthcare providers [49, 50]. Knee injury is a well-known risk factor for the development of early knee osteoarthritis [51, 52]. Also, it increases the risk for another new knee injury in the following year [53]. It should be of concern of the healthcare providers and researchers to study and implement factors that can decrease the rate of these injuries, to prevent the long-term physical and economic consequences.

## Conclusion

Knee injuries are common in our society. Soccer was the predominant sport associated with knee injuries, most of these injuries were treated conservatively. Stretching and warming up did not help with reducing the incidence of injuries. We need to raise the awareness and knowledge about these injuries. A thorough research for prevention methods and factors need to be investigated and studied to know and apply effective measures in decreasing the rate of these injuries and its complications. In addition, further research is needed to estimate the prevalence among females of such injuries in our population and whether they have different patterns of injuries as compared to males.

## Data Availability

The datasets used during the current study are available from the corresponding author on reasonable request.

## Abbreviations

ACL: Anterior cruciate ligament
PCL: Posterior cruciate ligament
MCL: Medial collateral ligament
LCL: Lateral collateral ligament
BMI: Body mass index
US: United States
